# The Effect of Personalization on Smartphone-Based Fall Detectors

**DOI:** 10.3390/s16010117

**Published:** 2016-01-18

**Authors:** Carlos Medrano, Inmaculada Plaza, Raúl Igual, Ángel Sánchez, Manuel Castro

**Affiliations:** 1 EduQTech, E.U. Politécnica de Teruel, University of Zaragoza, c/Atarazana 2, 44003 Teruel, Spain; inmap@unizar.es (I.P.); rigual@unizar.es (R.I.); asespilez@hotmail.com (A.S.); 2 Electrical, and Computer Engineering Department, Spanish University for Distant Education (UNED), C/ Juan del Rosal, 12, 28040 Madrid, Spain; mcastro@ieec.uned.es

**Keywords:** fall detection, smartphone, personalization, novelty detection

## Abstract

The risk of falling is high among different groups of people, such as older people, individuals with Parkinson's disease or patients in neuro-rehabilitation units. Developing robust fall detectors is important for acting promptly in case of a fall. Therefore, in this study we propose to personalize smartphone-based detectors to boost their performance as compared to a non-personalized system. Four algorithms were investigated using a public dataset: three novelty detection algorithms—Nearest Neighbor (NN), Local Outlier Factor (LOF) and One-Class Support Vector Machine (OneClass-SVM)—and a traditional supervised algorithm, Support Vector Machine (SVM). The effect of personalization was studied for each subject by considering two different training conditions: data coming only from that subject or data coming from the remaining subjects. The area under the receiver operating characteristic curve (AUC) was selected as the primary figure of merit. The results show that there is a general trend towards the increase in performance by personalizing the detector, but the effect depends on the individual being considered. A personalized NN can reach the performance of a non-personalized SVM (average AUC of 0.9861 and 0.9795, respectively), which is remarkable since NN only uses activities of daily living for training.

## 1. Introduction

Falls are an important public health concern, especially for some groups of people. It is estimated that about one-third of those over 65 years old experience one or more falls every year, but this fraction is even higher among the oldest ones [1]. Many fallers will suffer from the consequences: hip fracture, head trauma or bruises. In addition, those aged 65 and over will become a larger share of the population. For instance, in the European Union, the share will increase from 17% in 2010 to 30% in 2060 [2]. Thus, the possibility of building robust fall detection systems is very attractive for them. Fall detectors have the potential to provide rapid assistance after a fall, reducing the time period that the person is lying on the floor, which is one of the main factors that determine the severity of a fall [3]. Fall detectors are especially interesting for older people living alone or in remote areas, given that, if a fall occurs, it is unlikely they can have quick assistance. Characteristics such as ubiquity, control over the device and automatic response are seen as advantages that promote the acceptation and use of this kind of technology [4,5]. Furthermore, it is worth noting that not only old people suffer from falls. Young adults with impaired gait and balance or medium-to-severe motor disability appear to be at increased risk of falling [6] and they are likely to accept technology.

Over the recent years, a number of Information and Communication Technologies (ICTs) have emerged aimed at fall prevention, fall detection and alarms for use in case of a fall. Fall detectors can be classified according to the sensor used: wearable sensors, ambient sensors (pressure, sound) or cameras [7,8]. Research on vision systems is a hot topic [9] but privacy remains an important concern from the view of older adults [5]. Wearable sensors are very popular because they measure the user's movements directly. Most of the proposed systems use accelerometers, sometimes with other sensors such as gyroscopes [10]. Since smartphones integrate these sensors and solve the problem of communication, they have become very popular for fall detection [9,11]. The first of such systems that discriminated falls from activities of daily living (ADL) were based on simple threshold algorithms, in which the values of the acceleration in peaks or valleys were used to trigger an alarm [12,13]. However, the performance of these algorithms degrades in real situations as shown in [14]. Thus, researchers are turning towards more sophisticated machine learning algorithms [15,16].

Some recent publications [9,11] show that smartphones can play an important role in fall detection and in the reduction of fear of falling. This is because they have become an everyday technology, can be programmed to give feedback and present a simple interface. In fact, the use of smartphones among older people is increasing [17]. Regarding the usability problems that may arise, fall detection applications can be programmed in such a way that they can override the normal operation of the elements of the smartphone that can represent a usability barrier for low-skilled users [18]. Thus, they can boost the factors that facilitate the adoption of technology by older people [5], such as usability, control, feedback and cost. One of the main advantages of smartphones is that they have the potential to detect a fall when they are worn normally, for example in a pocket. The challenge is to find a technique able to detect a fall without any placement restriction, which is much more challenging than detecting a fall when the device is worn in a predefined part of the body [16,19]. In addition, the broad adoption of smartphones can avoid the stigmatization of wearing a special device, which in some cases caused people to feel more vulnerable [20]. However, it is likely that reducing the fear of falling is related to the user's perception of reliability and accuracy. In this regard there are still a number of issues:

- The characteristics of sensors integrated in smartphones are not as good as their counterparts in dedicated devices. Existing fall datasets are recorded using smartphones with accelerometers of a range of only ±2 g (g = gravity acceleration). Modern devices include accelerometers with higher ranges (3–16 g), so this problem may be mitigated in the future.
- Battery life is still a limitation and unnecessary computation in the phone should be avoided.
- The lack of real-world fall data is common to many fall detector studies. Most studies are based on laboratory falls performed by young or mature healthy people. Few studies include real-life data, but the number of falls is still low and there is no public dataset.
- Instead of building a generic fall detector, it would be interesting to adapt the detector to each user, since the movements and requirements depend on the context: a fall in the construction industry, a fall from a bed in a hospital, a fall of an older person, a fall of a person suffering from movement disorders, *etc.*

In this paper, we focus on the personalization of fall detectors and, to some extent, on the avoidance of dealing with real-world fall data. Few papers deal with the adaptation of fall detection. In [12], the threshold depends on user-provided parameters such as height, weight and level of activity. In [21], a real-time unit attached to the user stores ADL and performs fall detection. When fall detection is not required (at nighttime or inactivity periods), the real-time unit sends the ADL to a non-real-time unit, where the threshold is changed accordingly. However, there is no report of improvement on performance. This contrasts with the impact of personalization in activity recognition highlighted in other studies [19,22,23]. Thus, it seems that personalization and adaptation are important issues in fall detection.

In our previous work [19], we proposed the use of novelty detection techniques [24,25,26] for fall detection. It is worth highlighting that these kinds of approaches differ from typical classification systems. In fact, they detect any abnormal movement that deviates from common user movements. Some of these techniques only rely on a normal class for training, which is ADL in our case. ADL can be recorded in real life, as opposed to in laboratory experimental falls. Therefore, they are good candidates for personalization, since they can be re-trained with the new data of a given user. We have shown that a nearest-neighbor algorithm (NN) can run comfortably in low-end smartphones and adapt to each user on the fly [27]. The purpose of the present paper is to go further into our research on personalized fall detectors. In particular, we have extended the study to more sophisticated novelty detector algorithms using the complete three-axial acceleration information. In addition, we have studied the effect of personalization not only on novelty detectors but also on the Support Vector Machine (SVM), which is a traditional supervised classifier that needs both ADL and falls for training. Personalization using real-world falls from a given person is unrealistic, but at least SVM can be adapted with new, true ADL from a given user recorded while he/she carries the phone.

## 2. Experimental Section

### 2.1. Dataset

We have used a public dataset of ADL and falls recorded with smartphones [19]. The dataset comprises about 8000 ADL and 500 falls from 10 people ranging from 20 to 42 years old. Although the number of subjects is limited, the amount of collected samples is well above most fall datasets [28]. Eight different types of falls are included: forward falls, backward falls, left- and right-lateral falls, syncope, sitting on empty air, falls using compensation strategies to prevent the impact, and falls with contact with an obstacle before hitting the ground. During the fall simulation, participants were asked to give one or two small steps or to stay and stand and then perform the fall activity, remaining lying after the fall. Falls were completed on a soft mattress in a laboratory environment. During the falls, participants wore a smartphone in both their two pockets (left and right). Each fall type was simulated three times for a total of about 50 fall samples per subject. Regarding the ADL study, ADL were taken while the volunteers carried the phone in a pocket while they performed their daily activities. Thus, the ADL study was carried out under real-life conditions, without the only indication of acting as they do usually. On average, about 800 ADL were collected from each subject. The phone was a Samsung Galaxy Mini. Its built-in accelerometer has a range of ±2 g. The study protocol was approved by the Ethical Committee for Clinical Research of Aragon (CEICA). All subjects received oral and written information about the study, and written informed consent was obtained from them.

In the continuous monitoring period, the ADL were obtained by finding maximum acceleration values in a given time, and then recording windows of 6 s around that maximum peak. Peaks under 1.5 g were disregarded. Falls were recorded in the same format. Thus, the public dataset consists of independent 6 s time windows with the peak in the central sample. In this paper, we have taken only the information of acceleration around the signal magnitude peak in a one-second time-window. Since the data were interpolated at 50 Hz, this implies that each ADL or fall was transformed into a 3 × 51 dimension vector, keeping the information of the three-axis acceleration. This is called a record henceforth. The algorithms were applied in this vector space. In the ideal case, it should be possible to detect falls based on short windows, since falls themselves are very short according to authors such as Noury *et al.* [29]. Our main goal was to focus on personalization, in which the key aspect is likely to be the shape of the acceleration depending on the way each person moves.

### 2.2. Algorithms and Their Evaluation

In this paper, we have tested three novelty detection algorithms (that only require ADL for training) and one supervised technique (that requires both ADL and falls for training). The novelty detection algorithms were: nearest neighbor (NN), local outlier factor (LOF), and One-Class Support Vector Machine (OneClass-SVM). All three algorithms only rely on ADL in the training set, thus they learn the normal behavior and detect any anomalous movement. We consider that these kinds of algorithms are the best suited for personalization, since they can be re-trained with new ADL recorded while a new user starts carrying the phone. In the off-line analysis presented in this paper, personalization implies training an algorithm using only data from a given subject, as explained below.

The three algorithms were based on the same concept of distance between two records. Given acceleration records A = {$a_{i}$, i = 1 ... T} and B = {$b_{i}$, i = 1 ... T}, where i is the time index, and T defines the length of the window (from −0.5 s to 0.5 s, the central peak time being 0), the distance between two records was defined as:

(1) $$d\left(A,B\right)=\;\sqrt{\underset{i}{\sum }{\left\|a_{i}-b_{i}\right\|}^{2}}$$

This distance is equivalent to the Euclidean distance in the 3 × 51 dimensional vector space, considering all the samples and the three axes of a record concatenated into a single vector. It is a measure of the differences between two acceleration shapes without focusing on particular points (peaks, valleys).

Then, given a set of N exemplars from a training set {**B**^j^, j = 1 … N}, the nearest neighbor distance of a new acceleration record, **C**, was defined as:

(2) $$d_{NN}\left(C\right)=min_{j}\;d\left(C,\;B^{j}\right)$$

Despite its simplicity, NN has been applied to many problems successfully. However, algorithms based on *d_NN_* are thought to perform poorly if the data has regions of varying densities. Thus, researchers have developed other algorithms that take into account the relative density of a given data instance to compute the anomaly score. A state-of-the art technique is LOF [30], in which the score is equal to the ratio of the average local density of the k nearest neighbors of the instance and the local density of the data instance itself. Specifically, the LOF score was based on the concepts of reachability distance and reachability density. If *d_kNN_(**C**)* is the distance to the k-nearest neighbor, then the reachability distance between two records was defined as [30]:

(3) $$d_{reach}\left(C,\;D\right)=\text{max}\left(\;d_{kNN}\left(C\right),\;d\left(C,D\right)\right)$$

By taking the maximum in the definition of the reachability distance, the statistical fluctuations of *d(**C**, **D**)* for records close to **C** are reduced. In addition, the reachability density was defined as [30]:

(4) $$\rho_{reach}\left(C\right)=\;\frac{\left|N_{k}\left(C\right)\right|}{\sum_{D\;\in N_{k}\left(C\right)}d_{reach}\left(C,D\right)}$$

where *N_k_(**C**)* is the k-neighborhood of record **C**, meaning the set of its k-nearest neighbors, and *|N_k_(**C**)|* its number. Then, the LOF score was defined as [30]:

(5) $$LOF\left(C\right)=\;\frac{\sum_{D\;\in N_{k}\left(C\right)}\frac{\rho_{reach}\left(D\right)}{\rho_{reach}\left(C\right)}\;}{\left|N_{k}\left(C\right)\right|}$$

Inside a tight cluster, the LOF score is expected to be close to 1, while it increases for outliers [30].

Finally, we have also used a more complex algorithm, OneClass-SVM [31]. This technique finds a hyperplane that tries to separate a fraction of the input data from the rest by the sign of the distance to the hyperplane (f(**C**) > 0 or f(**C**) < 0). The technique is based on concepts similar to those found in SVM (maximum margin, kernel trick, support vectors, *etc.*). Schölkopf *et al.* [31] demonstrated that the decision function estimates the support of the underlying probability distribution, p(**C**), from which input data are supposed to be sampled. That is, f(**C**) > 0 in a region S of the input space which concentrates most of the probability mass function. The algorithm includes a parameter, ν, which is an upper bound to the fraction of outliers.

To compare with a traditional supervised algorithm, we have selected the Support Vector Machine (SVM) [32]. This algorithm needs both ADL and falls for training. As its OneClass counterpart, SVM finds a hyperplane that separates two regions [32]. Thanks to the kernel trick, this hyperplane can be in a space different from the original input feature space, leading to a better discrimination. The algorithm has a constant C, which is a penalization term for misclassified records. In both cases, SVM and OneClass-SVM, the popular Radial Basis Function Kernel was selected:

(6) k(A, B)=exp(− γ d(A, B))

where γ is a constant and d(A, B) is the distance defined in Equation (1).

**Figure 1:**
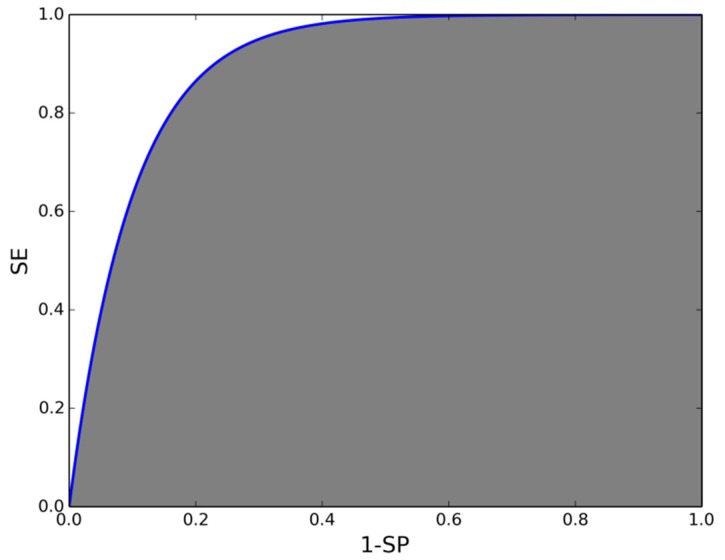
Schematic representation of the ROC curve (blue line). The gray area is the AUC. For a perfect detector this curve has an inverted-L shape, and the area under it covers the whole graph.

The figure of merit we have chosen to compare the algorithms is the area under the receiver operating characteristic curve (AUC). This curve represents the sensitivity (SE) *versus* the False Positive Ratio—equivalent to 1-SP, where SP is the specificity. The AUC is the area under this curve. For a perfect detector AUC has a value of 1. The closest a detector’s AUC is to 1, the better the detection is (see an example representation of this curve in Figure 1). This is a convenient and typical parameter for novelty detectors such as NN and LOF, because they do not provide a decision boundary by themselves. Nevertheless, there exists a threshold that can be changed, leading the detector to two extreme regimes: either every input is detected as a fall (SE = 1.0, SP = 0.0) or no movement triggers the alarm (SE = 0.0, SP = 1.0). The threshold is applied to the distance to the nearest neighbor in NN, to the LOF score or to the distance to the hyperplane found in SVM or OneClass-SVM.

Although AUC is the primary figure of merit in this paper, values of SE and SP will be provided since they are more intuitive and common in previous studies. For that purpose, the point in the curve that maximized the geometric mean of SE and SP, $\sqrt{SE\;SP}$, was selected, and the specific values of SE and SP were also obtained.

It is worth explaining that this study has been carried out in two steps.

- First, we have compared the novelty detectors between them and selected the best one using a conventional cross-validation strategy (Section 3.1).
- Then, the selected novelty detector (NN) has been compared with SVM subject by subject, thus trying to study personalization, which is the main focus of this paper (Section 3.2).

For the comparison between the algorithms in the first step, we have used a conventional 10-fold cross-validation, dividing the entire dataset in 10 parts, and taking one of them for validation each time and the rest for training.

**Figure 2:**
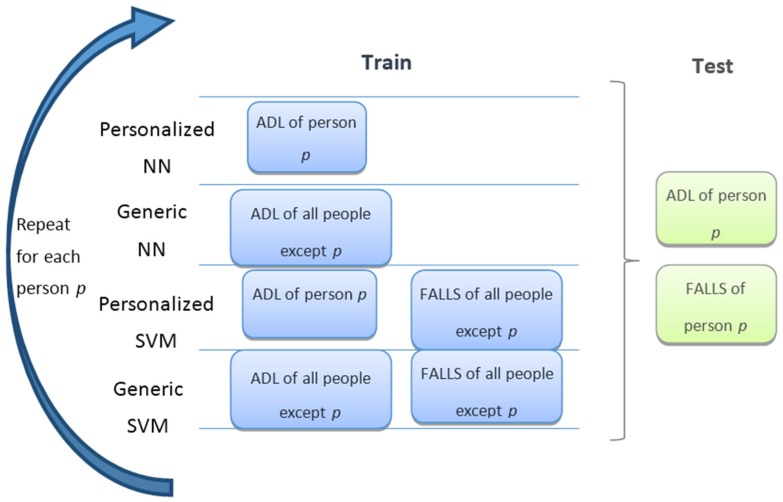
Schematic view of test and training to study the effect of personalization. Boxes of different colors with the same label represent different parts of a dataset. For each subject, the process was repeated 10 times for cross-validation.

In the second step, the study of the influence of the personalization has been conducted by comparing generic and personalized versions of NN and SVM. This comparison was repeated for each subject. It is important to highlight that in the personalized version of the algorithms, the training dataset included only ADL from the selected subject, while in the generic detector an equal number of ADL from the rest of the subjects were used. The validation set consisted of data from the selected subject. This is schematically shown in Figure 2. Even though it is not shown in the figure for simplicity, a cross-validation was also performed for each subject by taking a different 10% of his/her data as a validation set in each run.

In any of the experiments, whenever two results were compared, a two-sided Wilcoxon test for related samples was applied.

The unknown parameters of the algorithms are the following: C and γ (SVM), ν and γ (OneClass-SVM), and k (LOF). They were set every time the model was trained, by selecting the best values after a grid search, using cross-validation in the training set to estimate performance [32]. That is, any training set was further split into two sets, S1 (90%) and S2 (10%). S1 was used to train the model and S2 to measure performance. This was repeated 10 times and the parameters that gave the best average performance were selected.

NN has no free parameters. As we will see below, it outperformed OneClass-SVM and LOF, which were discarded in the first stage of the study. Thus, in the personalization stage of the study only the parameters of SVM were required to be found.

The algorithms NN and LOF were implemented in Python with our own code, while for OneClass-SVM and SVM we used the implementation of the scikit-learn package [33] (version 10.0, which comes as a package for Ubuntu 12.04). Statistical analyses were done with R [34].

## 3. Results

### 3.1. Comparison between Novelty Detectors

Table 1 presents the comparison between novelty detectors based on AUC. NN showed better performance than LOF and OneClass-SVM, although the difference with LOF is not statistically significant. Table 2 presents values of SE and SP. NN is again the best in terms of $\sqrt{SE\;SP}$. Thus, for further investigation, we focused only on NN.

**Table 1:** Comparison between novelty detectors. AUC values are given as means and standard deviations. *p*-values were obtained with a two-sided Wilcoxon test.

**NN**	**LOF**	**OneClass-SVM**
0.9809 ± 0.0028	0.9784 ± 0.0048	0.9644 ± 0.0051
**Differences**		
**NN-LOF**	**NN-OneClass-SVM**	**LOF-OneClass-SVM**
0.0025 ± 0.0066	0.0165 ± 0.0039	0.0140 ± 0.0083
***p*-value**		
0.27	<0.01	<0.01

**Table 2:** Values of SE, SP and their geometric mean for three different novelty detectors. Values are given as means and standard deviations.

	NN	LOF	OneClass-SVM
**SE**	0.9541 ± 0.0064	0.9622 ± 0.0189	0.9156 ± 0.0123
**SP**	0.9484 ± 0.0059	0.9364 ± 0.0199	0.9417 ± 0.0083
$\sqrt{SE\;SP}$	0.9512 ± 0.0046	0.9491 ± 0.0142	0.9285 ± 0.0091

### 3.2. Personalization

The average values of AUC for each person are presented in Table 3. About 97.5% of the cells are higher than 0.90, and 92.5% are higher than 0.95. Averaged over people, the best classifiers are, in decreasing order, personalized SVM (PSVM), personalized NN (PNN), generic SVM (GSVM) and generic NN (GNN).

**Table 3:** AUC values (mean and standard deviation) for the algorithm variants: Personalized NN (PNN), generic NN (GNN), personalized SVM (PSVM) and generic SVM (GSVM). The last row is the average over subjects.

User	Algorithm			
	PNN	GNN	PSVM	GSVM
**1**	0.9770 ± 0.0058	0.9463 ± 0.0097	0.9881 ± 0.0039	0.9667 ± 0.0063
**2**	0.9877 ± 0.0101	0.9829 ± 0.0092	0.9929 ± 0.0069	0.9905 ± 0.0079
**3**	0.9900 ± 0.0060	0.9713 ± 0.0059	0.9948 ± 0.0043	0.9912 ± 0.0067
**4**	0.9878 ± 0.0067	0.9744 ± 0.0064	0.9930 ± 0.0053	0.9845 ± 0.0046
**5**	0.9760 ± 0.0074	0.9720 ± 0.0069	0.9766 ± 0.0068	0.9745 ± 0.0095
**6**	0.9903 ± 0.0072	0.8460 ± 0.0341	0.9967 ± 0.0030	0.9405 ± 0.0127
**7**	0.9780 ± 0.0108	0.9554 ± 0.0140	0.9870 ± 0.0065	0.9905 ± 0.0028
**8**	0.9863 ± 0.0032	0.9591 ± 0.0048	0.9957 ± 0.0054	0.9724 ± 0.0037
**9**	0.9909 ± 0.0099	0.9894 ± 0.0087	0.9949 ± 0.0072	0.9952 ± 0.0054
**10**	0.9967 ± 0.0009	0.9855 ± 0.0036	0.9942 ± 0.0014	0.9892 ± 0.0023
**Mean**	0.9861 ± 0.0023	0.9582 ± 0.0042	0.9914 ± 0.0017	0.9795 ± 0.0022

The detailed comparisons between pairs of algorithms are shown in bar diagrams, where we compare, for each subject, the difference in AUC.

**Figure 3:**
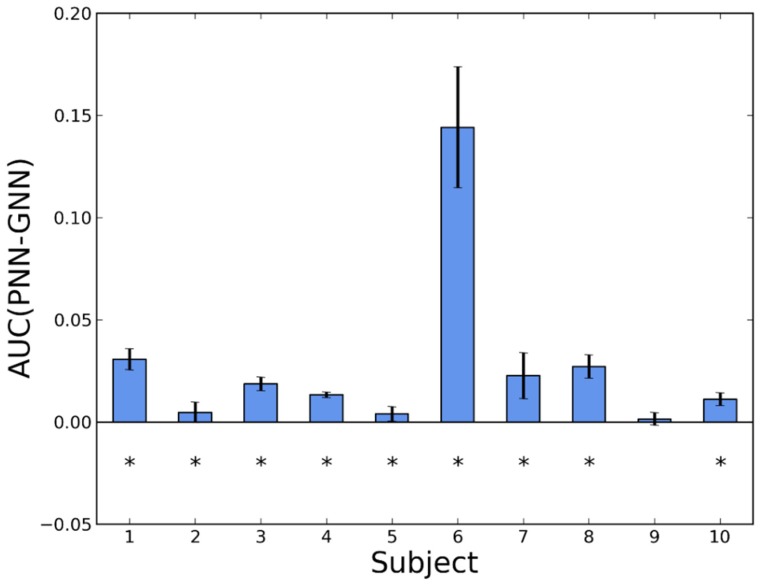
Difference in AUC between a personalized and a generic version of NN (PNN and GNN, respectively). Whiskers represent standard deviations. Asterisks indicate *p*-values < 0.05 in a Wilcoxon test.

In Figure 3 we show the effect of personalization for NN. The difference is in favor of the personalized detector for the 10 volunteers. In nine of them, the difference is statistically significant. Taking into account the information from all the subjects, we have estimated the global mean improvement in AUC as 0.0278 ± 0.0034.

**Figure 4:**
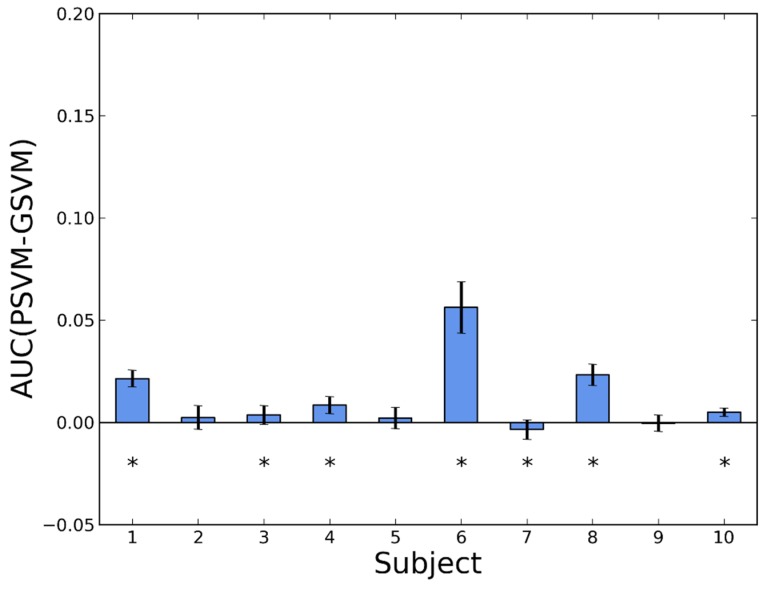
Difference in AUC between a personalized and a generic version of SVM (PSVM and GSVM, respectively). Whiskers represent standard deviations. Asterisks indicate *p*-values < 0.05 in a Wilcoxon test.

In Figure 4 the effect of personalization in SVM is shown. In this case, the personalization does not always help. For eight people, the performance of the personalized detector is better, but for the remaining two it is not, although with small differences. If we only consider the results with *p*-value < 0.05, these numbers reduce to six and one, respectively. The estimated average of the improvement was 0.0119 ± 0.0019.

**Figure 5:**
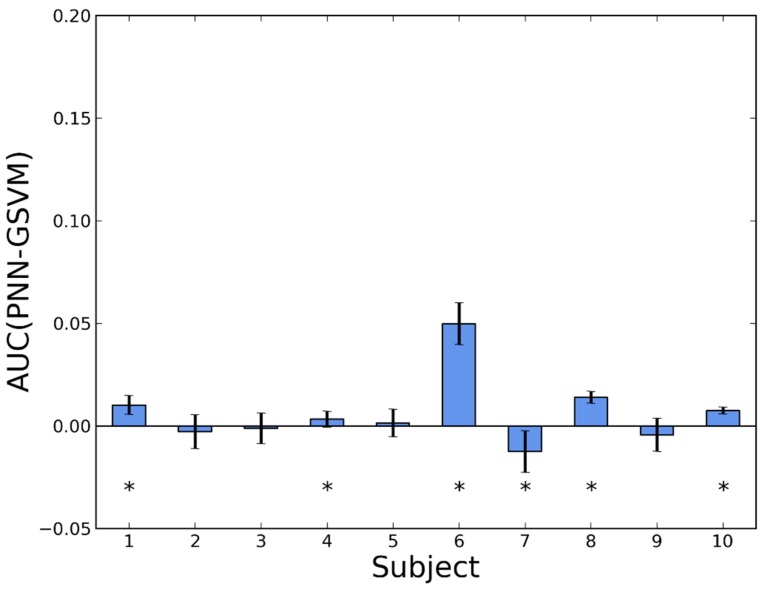
Difference in AUC between a personalized NN (PNN) and a generic SVM (GSVM). Whiskers represent standard deviations. Asterisks indicate *p*-values < 0.05 in a Wilcoxon test.

In Figure 5 we compare PNN with GSVM. In this case, the results are much more similar. For six people PNN shows better performance and for four people GSVM provides higher detection rates. After rejecting the unclear cases, the numbers reduce to five and one, respectively. Averaging over people, PNN is better by 0.0066 ± 0.0022.

**Figure 6:**
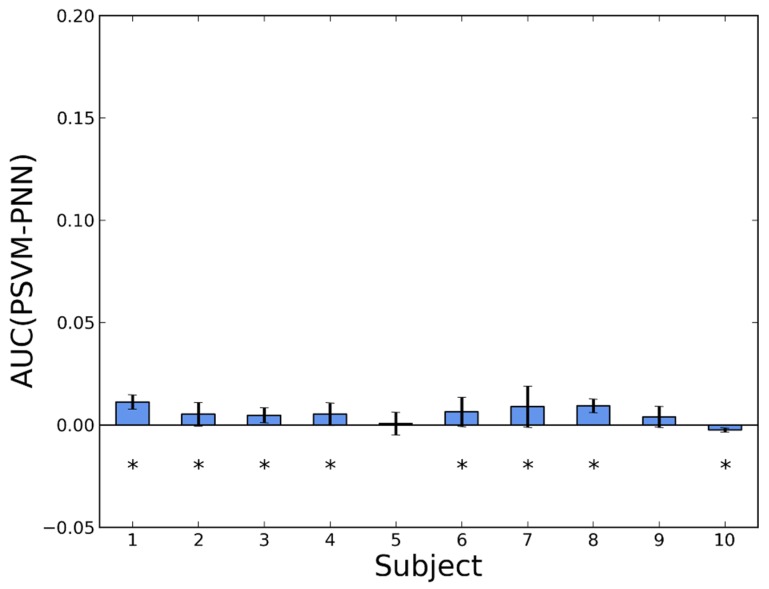
Difference in AUC between the personalized versions of SVM (PSVM) and NN (PNN). Whiskers represent standard deviations. Asterisks indicate *p*-values < 0.05 in a Wilcoxon test.

Finally, in Figure 6 we compare the two personalized versions of the algorithms (PNN and PSVM). Except for one person, PSVM is better, and only in two cases the *p*-value was higher than 0.05. On average, PSVM outperformed PNN by 0.0053 ± 0.0018.

Values of SE, SP and their geometric mean are shown in Table 4. For simplicity, each cell contains the average value of the corresponding cross-validation, without standard deviation. About 97% of the cells contain values higher than 0.90, and 75% contain values higher than 0.95. The average values for the personalized detectors are near 0.97 (last row, bold face). To summarize the comparison between pairs of classifiers, Table 5 shows the number of subjects for whom one classifier outperformed the other. For instance, the last row presents the geometric mean of SE and SP. PNN is better than GNN for 10 people. The personalization also helps when comparing PSVM and GSVM (nine *versus* one). The comparison between PNN and GSVM yields a tie (five *versus* five), while PNN is worse than PSVM in general (one *versus* nine).

**Table 4:** Values of SE, SP and their geometric mean for each person and different versions of the classifiers. The last row is the average value over subjects, where personalized values are highlighted in bold.

User	PNN			GNN			PSVM			GSVM		
	SE	SP	$\sqrt{SE\;SP}$	SE	SP	$\sqrt{SE\;SP}$	SE	SP	$\sqrt{SE\;SP}$	SE	SP	$\sqrt{SE\;SP}$
**1**	0.9216	0.9817	0.9511	0.8980	0.9656	0.9311	0.9510	0.9655	0.9580	0.9333	0.9742	0.9535
**2**	0.9906	0.9682	0.9792	0.9774	0.9429	0.9599	1.0000	0.9627	0.9811	0.9962	0.9594	0.9776
**3**	0.9737	0.9740	0.9738	0.9105	0.9625	0.9360	0.9816	0.9750	0.9781	0.9763	0.9625	0.9693
**4**	0.9648	0.9689	0.9667	0.9500	0.9661	0.9579	0.9907	0.9605	0.9754	0.9648	0.9717	0.9681
**5**	0.9154	0.9316	0.9230	0.9019	0.9377	0.9186	0.9346	0.9304	0.9322	0.9212	0.9450	0.9325
**6**	0.9880	0.9728	0.9803	0.9560	0.7990	0.8735	1.0000	0.9852	0.9926	0.9960	0.8365	0.9126
**7**	0.9815	0.9577	0.9694	0.9704	0.9240	0.9467	0.9815	0.9859	0.9837	0.9759	0.9690	0.9724
**8**	0.9623	0.9878	0.9749	0.9113	0.9443	0.9274	0.9811	0.9895	0.9853	0.9509	0.9827	0.9667
**9**	0.9882	0.9772	0.9826	0.9824	0.9590	0.9703	0.9980	0.9863	0.9921	0.9863	0.9795	0.9828
**10**	0.9787	0.9950	0.9868	0.9574	0.9825	0.9699	0.9787	0.9925	0.9856	0.9468	0.9925	0.9693
**Mean**	0.9665	0.9715	0.9688	0.9415	0.9384	0.9391	0.9797	0.9734	0.9764	0.9648	0.9573	0.9605

**Table 5:** Summary of the comparison with respect to SE, SP and $\sqrt{SE\;SP}$. For each pair comparison, a cell contains the number of subjects for whom the member of the pair is better for SE, SP or their geometric mean.

	PNN *vs.* GNN		PSVM *vs.* GSVM		PNN *vs.* GSVM		PNN *vs.* PSVM	
**SE**	10	0	10	0	4	6	0	10
**SP**	9	1	6	4	6	4	5	5
$\sqrt{SE\;SP}$	10	0	9	1	5	5	1	9

## 4. Discussion

In the comparison between novelty detectors, three methods have been considered: the basic NN and two state-of-the-art algorithms, LOF and OneClass-SVM. However, NN has shown to be the most suitable one. The difference with LOF is not significant, but there is no reason to select LOF. NN is conceptually and computationally simpler, there is no need to tune any parameter and it can be implemented in smartphones easily. Therefore, this result is reassuring. Some reasons can be suggested to explain it. LOF is based on a local definition of outliers since it is based on ratios of neighborhood densities. It is suitable when there are clusters of different densities. However, it did not improve the results. This can be due to the fact that most ADL are repeated several times per day, so that there is enough numbers of similar ADL in the dataset and, therefore, the concept of different densities is not necessary. OneClass-SVM has a solid theoretical foundation. However, it is sensitive to outliers that can change the hyperplane position to a high degree. Some robust alternatives have been proposed [35]. It is also possible that the features used are good for an NN classifier, since they give a measure of acceleration shape difference directly, but are not suitable for density estimation in OneClass-SVM. Thus, features obtained from a more refined processing of the raw input signal could be tested.

The results of NN (0.9809 ± 0.0028) are better than those obtained in our previous work (0.9554 ± 0.0052) (see Table 1 in [19]). This is because, in the present work, we have used three-axis acceleration values and not only the signal magnitude acceleration as we did in [19]. By taking the values along the three axes, we are including, in some way, information about the orientation. Thus, the orientation of the phone is also an important feature for fall detection, even though the records in the dataset were obtained with the phone in the pocket, where it is only loosely fixed and can be rotated in several angles. This three-dimensional (3D) information is lost if only the signal magnitude was used. We have checked that the improvement is also true for all the other algorithms (LOF, OneClass-SVM and SVM).

With respect to personalization itself, the results show a trend towards improving the performance of the detector. In average, the personalized detector is clearly better. This is also true when the results are seen person by person in NN. Personalization also improves SVM globally, even though the improvement is lower and the results, person by person, are less strong. This could be because the generic baseline SVM is already very good in terms of AUC, thus there is less room for improvement and the differences are lower. The conclusion is in favor of personalization not only because there are more cases in which it is better, but also because the improvement is also higher and stands out for some subjects. Thus, it could be said that personalization is a worst-case improvement option. In NN, the average difference in AUC due to personalization is 0.0278, but the worst cases are 0.1422 and 0.0307, while in SVM the mean difference is 0.0119, but improvement in the worst cases are 0.0562 and 0.0233. Thus, for some people it would be more important to adapt the system. It would be wise to extend the study with more volunteers covering a wider range of fall contexts, especially to clarify the results in SVM. The improvement due to personalization is also found in SE, SP and $\sqrt{SE\;SP}$, with similar conclusions. Although the improvement in the figures of merit could appear to be modest, it can be important for end users. For instance, the average value of SP in NN improves from 0.9384 to 0.9715 (last row of Table 4), which means reducing the annoying false alarms by a factor of (1 − 0.9384)/(1 − 0.9715) = 2.16.

In regard to the comparison between PNN and GSVM, it is not possible to provide a clear conclusion at the moment. The results are very similar in terms of number of people for whom one algorithm is the best; on average, AUC and $\sqrt{SE\;SP}$ of PNN are higher*.* Anyway, this is a result to be highlighted: a novelty detector (which only needs ADL in the training phase) can reach a performance similar to that of a traditional supervised algorithm (which requires both ADL and falls for training). In addition, PNN is conceptually far simpler than GSVM and the personalization can be done on the fly in a smartphone [27]. It is worth pointing out that PNN is only based on ADL, thus it relies only on true data, while GSVM relies partially on laboratory data (falls), which may be different from real fall data [36]. Of course, the ultimate test for fall detectors must be done with real-world falls, but at least we have proven that a novelty detector can be a competitive system with the currently available data.

Finally, the comparison between PNN and PSVM shows that PSVM is better in most people. Nevertheless, the complexity of re-training in the phone is very different. NN is a pure data-driven method without an actual “training algorithm”. It is based on a set of exemplars from which the nearest neighbor is selected. The set could be stored in an ADL table, which would be updated as the user carries the phone and new movements are registered [27]. After a few days (two to three days for young people), the table is fully updated and, thus, adapted to the user. Over the first days, detection of falls could be more restrictive in order to avoid false alarms until the ADL table is fully updated. On the other hand, personalizing SVM in the phone would imply implementing the solution of a quadratic programming problem in the device [32], which is not so easy. Another alternative would be to send the new ADL to a server, re-train the classifier in the server, and send back the SVM model to the phone (support vectors, weights and bias).

Additionally, we should state that our work has some limitations. The dataset we have used corresponds to young or mature people but all of them are healthy subjects and registered with low-end smartphones (accelerometer range ±2 g). It would be interesting to compare people with different diseases or gait conditions. Using accelerometers with a larger range would allow testing a personalization of threshold-based algorithms since more restrictive peak or valley detection can help to reduce false alarms without any further processing. With respect to the detectors, we have used raw acceleration values of a short time window. A more intelligent feature extraction and inclusion of information about an extended time window could improve performance. Checking for inactivity or for change in orientation after the fall are typical solutions to avoid false alarms. The combination of several methods is also a line for future research. An intrinsic limitation of novelty detectors must also be acknowledged: they detect any kind of abnormal movements, not only falls. Thus, some rare movements could trigger the alarm as well.

In conclusion, this paper has shown that there is a general trend towards the increase in performance by personalizing a fall detector, and that a system which only uses ADL for training can obtain the performance of a system which requires both ADL and falls. This represents a change of paradigm in the traditional way in which a fall detection study is conceived.
